# Supplementary material for: Exercise-induced increase in blood-based brain-derived neurotrophic factor (BDNF) in people with multiple sclerosis: A systematic review and meta-analysis of exercise intervention trials
Source: PLoS One. 2022 Mar 3;17(3):e0264557. doi: 10.1371/journal.pone.0264557 (PMC8893651; doi:10.1371/journal.pone.0264557)
Supplement: S5 Fig — (DOCX) [file pone.0264557.s006.docx]

**Supplementary Figure S5. Results of Sensitivity analysis (leave-one-out analysis) of the meta-analysis (Influence Diagnostics)**

**
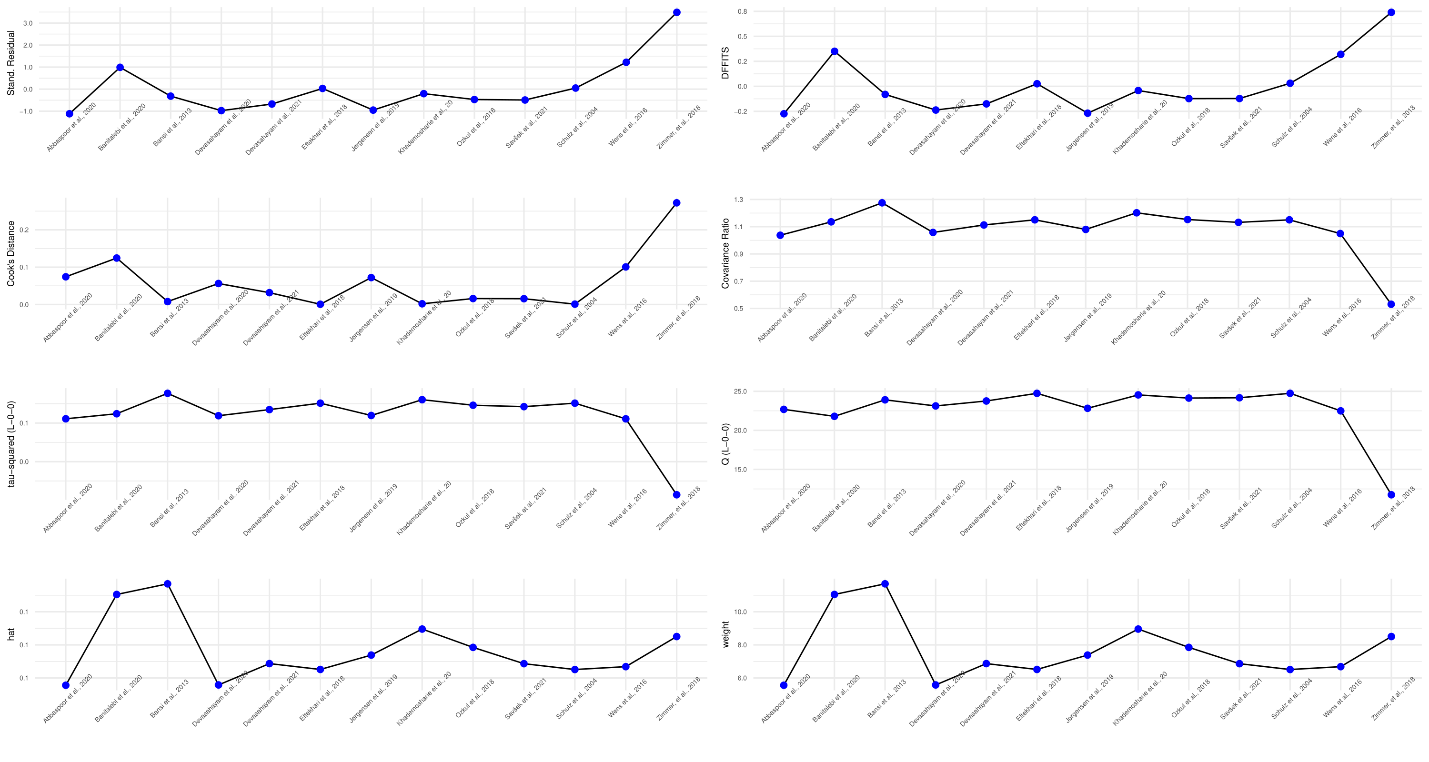
**
