# Supplementary material for: Exercise-induced increase in blood-based brain-derived neurotrophic factor (BDNF) in people with multiple sclerosis: A systematic review and meta-analysis of exercise intervention trials
Source: PLoS One. 2022 Mar 3;17(3):e0264557. doi: 10.1371/journal.pone.0264557 (PMC8893651; doi:10.1371/journal.pone.0264557)

**Supplementary Figure S6. Results of Sensitivity analysis (leave-one-out analysis) of the meta-analysis (I2 and effect size plot)**


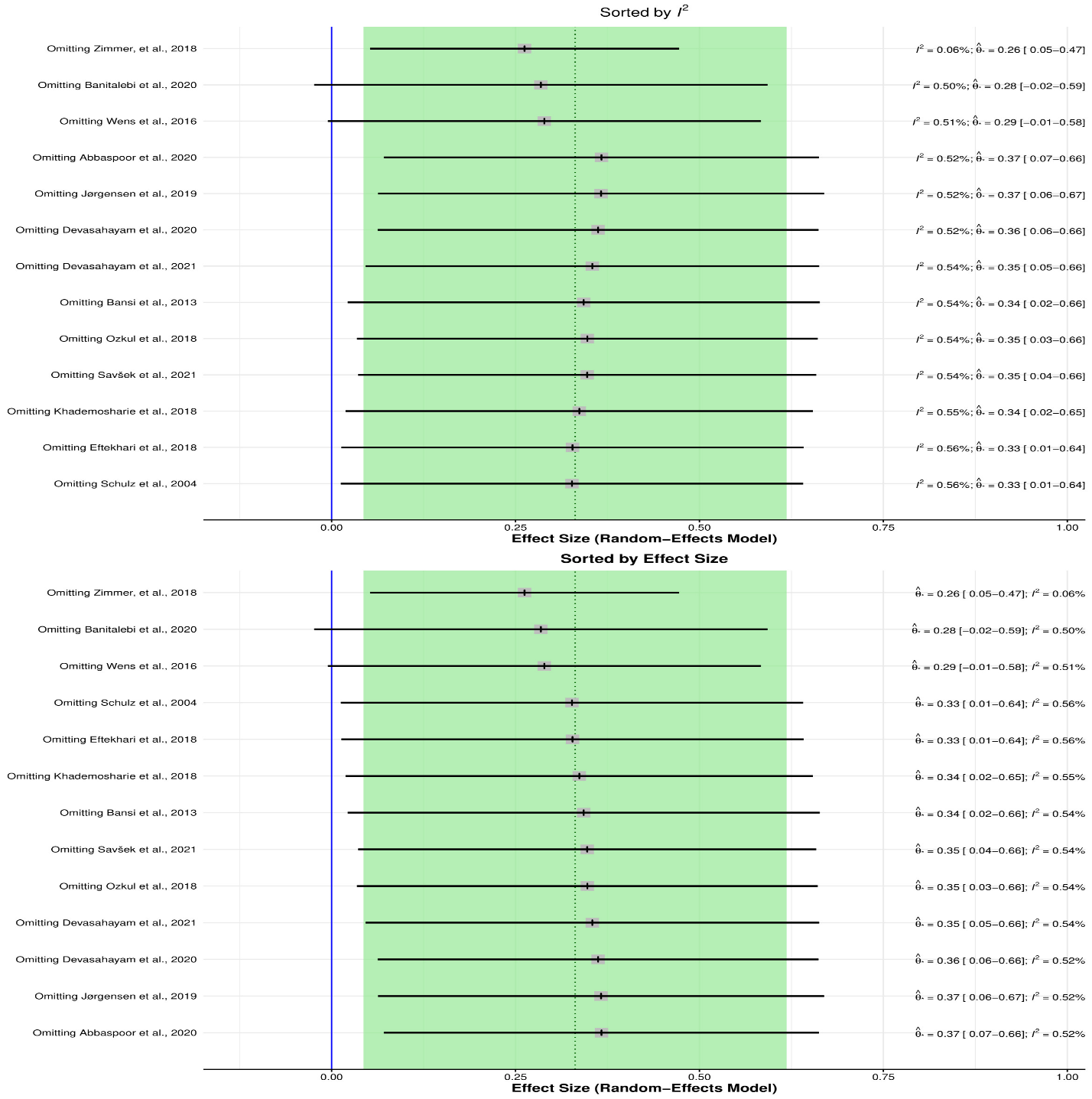

Supplement: S6 Fig — (DOCX) [file pone.0264557.s007.docx]
