# Supplementary material for: Exercise-induced increase in blood-based brain-derived neurotrophic factor (BDNF) in people with multiple sclerosis: A systematic review and meta-analysis of exercise intervention trials
Source: PLoS One. 2022 Mar 3;17(3):e0264557. doi: 10.1371/journal.pone.0264557 (PMC8893651; doi:10.1371/journal.pone.0264557)
Supplement: S1 Appendix — (DOCX) [file pone.0264557.s008.docx]

**Supplementary material**

**Title: Exercise-induced increase in blood-based brain-derived neurotrophic factor (BDNF) in people with multiple sclerosis: a systematic review and meta-analysis of exercise intervention trials**

**Authors:** Parnian Shobeiri, Amirali Karimi, Sara Momtazmanesh, Antônio L. Teixeira, Charlotte E. Teunissen, Erwin E.H. van Wegen, Mark A. Hirsch, Nima Rezaei*, Mir Saeed Yekaninejad*

***Corresponding authors:**

- Nima Rezaei, MD, Ph.D., Research Center for Immunodeficiencies, Children’s Medical Center, Dr. Gharib St, Keshavarz Blvd, Tehran, Iran, E-mail: [rezaei_nima@yahoo.com](mailto:rezaei_nima@yahoo.com)
- Mir Saeed Yekaninejad, PhD: Department of Epidemiology and Biostatistics, School of Public Health, Tehran University of Medical Sciences, Tehran, Iran, E-mail: [yekaninejad@yahoo.com](mailto:yekaninejad@yahoo.com)

***Table of Contents***

MEDLINE (PubMed interface)2

EMBASE4

Cochrane Central Register of Controlled Trials5

Physiotherapy Evidence Database6

1. **Search Strategies**
   1. **MEDLINE (PubMed interface)**

| #1 | (Sclerosis, Multiple[Title/Abstract]) OR (Sclerosis, Disseminated[Title/Abstract])) OR (Disseminated Sclerosis[Title/Abstract])) OR (MS[Title/Abstract])) OR (Multiple Sclerosis, Acute Fulminating[Title/Abstract])) OR (Multiple Sclerosis[Title/Abstract])) OR (Multiple Sclerosis[MeSH Terms])) OR (Multiple Sclerosis, Relapsing-Remitting[MeSH Terms])) OR (Multiple Sclerosis, Relapsing-Remitting[Title/Abstract])) OR (Multiple Sclerosis, Relapsing Remitting[Title/Abstract])) OR (Remitting-Relapsing Multiple Sclerosis[Title/Abstract])) OR (Multiple Sclerosis, Remitting-Relapsing[Title/Abstract])) OR (Remitting Relapsing Multiple Sclerosis[Title/Abstract])) OR (Relapsing-Remitting Multiple Sclerosis[Title/Abstract])) OR (Relapsing Remitting Multiple Sclerosis[Title/Abstract])) OR (Multiple Sclerosis, Acute Relapsing[Title/Abstract])) OR (Acute Relapsing Multiple Sclerosis[Title/Abstract])) OR (Multiple Sclerosis, Chronic Progressive[MeSH Terms])) OR (Multiple Sclerosis, Chronic Progressive[Title/Abstract])) OR (Chronic Progressive Multiple Sclerosis[Title/Abstract])) OR (Multiple Sclerosis, Progressive Relapsing[Title/Abstract])) OR (Multiple Sclerosis, Remittent Progressive[Title/Abstract])) OR (Remittent Progressive Multiple Sclerosis[Title/Abstract])) OR (Progressive Relapsing Multiple Sclerosis[Title/Abstract])) OR (Multiple Sclerosis, Secondary Progressive[Title/Abstract])) OR (Secondary Progressive Multiple Sclerosis[Title/Abstract])) OR (Multiple Sclerosis, Primary Progressive[Title/Abstract])) OR (Primary Progressive Multiple Sclerosis[Title/Abstract])) |
| --- | --- |
| #2 | (Brain-Derived Neurotrophic Factor[MeSH Terms]) OR (Brain-Derived Neurotrophic Factor[Title/Abstract])) OR (Brain Derived Neurotrophic Factor[Title/Abstract])) OR (Factor, Brain-Derived Neurotrophic[Title/Abstract])) OR (Neurotrophic Factor, Brain-Derived[Title/Abstract])) OR (BDNF[Title/Abstract]))) |
| #3 | (Exercise[MeSH Terms]) OR (Exercise[Title/Abstract])) OR (Exercises[Title/Abstract])) OR (Physical Activity[Title/Abstract])) OR (Activities, Physical[Title/Abstract])) OR (Activity, Physical[Title/Abstract])) OR (Physical Activities[Title/Abstract])) OR (Exercise, Physical[Title/Abstract])) OR (Exercises, Physical[Title/Abstract])) OR (Physical Exercise[Title/Abstract])) OR (Physical Exercises[Title/Abstract])) OR (Acute Exercise[Title/Abstract])) OR (Acute Exercises[Title/Abstract])) OR (Exercise, Acute[Title/Abstract])) OR (Exercises, Acute[Title/Abstract])) OR (Exercise, Isometric[Title/Abstract])) OR (Exercises, Isometric[Title/Abstract])) OR (Isometric Exercises[Title/Abstract])) OR (Isometric Exercise[Title/Abstract])) OR (Exercise, Aerobic[Title/Abstract])) OR (Aerobic Exercise[Title/Abstract])) OR (Aerobic Exercises[Title/Abstract])) OR (Exercises, Aerobic[Title/Abstract])) OR (Exercise Training[Title/Abstract])) OR (Exercise Trainings[Title/Abstract])) OR (Training, Exercise[Title/Abstract])) OR (Trainings, Exercise[Title/Abstract])) |
| #4 | **#1 AND #2 AND #3**  (((((((((((((((((((((((((((((Sclerosis, Multiple[Title/Abstract]) OR (Sclerosis, Disseminated[Title/Abstract])) OR (Disseminated Sclerosis[Title/Abstract])) OR (MS[Title/Abstract])) OR (Multiple Sclerosis, Acute Fulminating[Title/Abstract])) OR (Multiple Sclerosis[Title/Abstract])) OR (Multiple Sclerosis[MeSH Terms])) OR (Multiple Sclerosis, Relapsing-Remitting[MeSH Terms])) OR (Multiple Sclerosis, Relapsing-Remitting[Title/Abstract])) OR (Multiple Sclerosis, Relapsing Remitting[Title/Abstract])) OR (Remitting-Relapsing Multiple Sclerosis[Title/Abstract])) OR (Multiple Sclerosis, Remitting-Relapsing[Title/Abstract])) OR (Remitting Relapsing Multiple Sclerosis[Title/Abstract])) OR (Relapsing-Remitting Multiple Sclerosis[Title/Abstract])) OR (Relapsing Remitting Multiple Sclerosis[Title/Abstract])) OR (Multiple Sclerosis, Acute Relapsing[Title/Abstract])) OR (Acute Relapsing Multiple Sclerosis[Title/Abstract])) OR (Multiple Sclerosis, Chronic Progressive[MeSH Terms])) OR (Multiple Sclerosis, Chronic Progressive[Title/Abstract])) OR (Chronic Progressive Multiple Sclerosis[Title/Abstract])) OR (Multiple Sclerosis, Progressive Relapsing[Title/Abstract])) OR (Multiple Sclerosis, Remittent Progressive[Title/Abstract])) OR (Remittent Progressive Multiple Sclerosis[Title/Abstract])) OR (Progressive Relapsing Multiple Sclerosis[Title/Abstract])) OR (Multiple Sclerosis, Secondary Progressive[Title/Abstract])) OR (Secondary Progressive Multiple Sclerosis[Title/Abstract])) OR (Multiple Sclerosis, Primary Progressive[Title/Abstract])) OR (Primary Progressive Multiple Sclerosis[Title/Abstract])) AND ((((((Brain-Derived Neurotrophic Factor[MeSH Terms]) OR (Brain-Derived Neurotrophic Factor[Title/Abstract])) OR (Brain Derived Neurotrophic Factor[Title/Abstract])) OR (Factor, Brain-Derived Neurotrophic[Title/Abstract])) OR (Neurotrophic Factor, Brain-Derived[Title/Abstract])) OR (BDNF[Title/Abstract]))) AND (((((((((((((((((((((((((((Exercise[MeSH Terms]) OR (Exercise[Title/Abstract])) OR (Exercises[Title/Abstract])) OR (Physical Activity[Title/Abstract])) OR (Activities, Physical[Title/Abstract])) OR (Activity, Physical[Title/Abstract])) OR (Physical Activities[Title/Abstract])) OR (Exercise, Physical[Title/Abstract])) OR (Exercises, Physical[Title/Abstract])) OR (Physical Exercise[Title/Abstract])) OR (Physical Exercises[Title/Abstract])) OR (Acute Exercise[Title/Abstract])) OR (Acute Exercises[Title/Abstract])) OR (Exercise, Acute[Title/Abstract])) OR (Exercises, Acute[Title/Abstract])) OR (Exercise, Isometric[Title/Abstract])) OR (Exercises, Isometric[Title/Abstract])) OR (Isometric Exercises[Title/Abstract])) OR (Isometric Exercise[Title/Abstract])) OR (Exercise, Aerobic[Title/Abstract])) OR (Aerobic Exercise[Title/Abstract])) OR (Aerobic Exercises[Title/Abstract])) OR (Exercises, Aerobic[Title/Abstract])) OR (Exercise Training[Title/Abstract])) OR (Exercise Trainings[Title/Abstract])) OR (Training, Exercise[Title/Abstract])) OR (Trainings, Exercise[Title/Abstract])) |

- 1. **EMBASE**

| #1 | ('multiple sclerosis'/exp OR 'ms' OR 'chariot disease' OR 'chronic progressive multiple sclerosis' OR 'disseminated sclerosis' OR 'insular sclerosis' OR 'multiple sclerosis' OR 'multiple sclerosis, chronic progressive' OR 'multiple sclerosis, relapsing-remitting' OR 'primary progressive multiple sclerosis' OR 'relapsing remitting multiple sclerosis' OR 'relapsing-remitting multiple sclerosis' OR 'sclerosis multiplex' OR 'sclerosis, disseminated' OR 'sclerosis, insular' OR 'sclerosis, multiple' OR 'secondary progressive multiple sclerosis') |
| --- | --- |
| #2 | ('brain derived neurotrophic factor'/exp OR 'bdnf' OR 'brain derived neurotrophic factor' OR 'brain-derived neurotrophic factor') |
| #3 | ('exercise'/exp OR 'biometric exercise' OR 'effort' OR 'exercise' OR 'exercise capacity' OR 'exercise performance' OR 'exercise training' OR 'exertion' OR 'fitness training' OR 'physical conditioning, human' OR 'physical effort' OR 'physical exercise' OR 'physical exertion' OR 'training'/exp OR 'army training' OR 'athletic training' OR 'athletic training program' OR 'athletic training programme' OR 'detraining' OR 'military training' OR 'physical training' OR 'sport specific training' OR 'technical training' OR 'training' OR 'training athlete' OR 'training course' OR 'training program' OR 'training programme' OR 'training, athletic' OR 'training, military' OR 'training, physical' OR 'resistance training'/exp OR 'resistance exercise' OR 'resistance exercise training' OR 'resistance training' OR 'strength training' OR 'weight bearing exercise') |
| #4 | **#1 AND #2 AND #3**  ('multiple sclerosis'/exp OR 'ms' OR 'chariot disease' OR 'chronic progressive multiple sclerosis' OR 'disseminated sclerosis' OR 'insular sclerosis' OR 'multiple sclerosis' OR 'multiple sclerosis, chronic progressive' OR 'multiple sclerosis, relapsing-remitting' OR 'primary progressive multiple sclerosis' OR 'relapsing remitting multiple sclerosis' OR 'relapsing-remitting multiple sclerosis' OR 'sclerosis multiplex' OR 'sclerosis, disseminated' OR 'sclerosis, insular' OR 'sclerosis, multiple' OR 'secondary progressive multiple sclerosis') AND ('brain derived neurotrophic factor'/exp OR 'bdnf' OR 'brain derived neurotrophic factor' OR 'brain-derived neurotrophic factor') AND ('exercise'/exp OR 'biometric exercise' OR 'effort' OR 'exercise' OR 'exercise capacity' OR 'exercise performance' OR 'exercise training' OR 'exertion' OR 'fitness training' OR 'physical conditioning, human' OR 'physical effort' OR 'physical exercise' OR 'physical exertion' OR 'training'/exp OR 'army training' OR 'athletic training' OR 'athletic training program' OR 'athletic training programme' OR 'detraining' OR 'military training' OR 'physical training' OR 'sport specific training' OR 'technical training' OR 'training' OR 'training athlete' OR 'training course' OR 'training program' OR 'training programme' OR 'training, athletic' OR 'training, military' OR 'training, physical' OR 'resistance training'/exp OR 'resistance exercise' OR 'resistance exercise training' OR 'resistance training' OR 'strength training' OR 'weight bearing exercise') |

- 1. **Cochrane Central Register of Controlled Trials (CENTRAL, Wiley interface)**

| #1 | MeSH descriptor: [Multiple Sclerosis] explode all trees | 3571 |
| --- | --- | --- |
| #2 | MeSH descriptor: [Brain-Derived Neurotrophic Factor] explode all trees | 314 |
| #3 | MeSH descriptor: [Brain-Derived Neurotrophic Factor] explode all trees | 25356 |
| #4 | #1 AND #2 AND #3 | 4 |

- 1. **Physiotherapy Evidence Database (PEDro)**

| #1 | Abstract & Title: | multiple sclerosis or MS |
| --- | --- | --- |
| #2 | Therapy: | no selection |
| #3 | Problem: | no selection |
| #4 | Body part: | no selection |
| #5 | Subdiscipline | no selection |
| #6 | Topic: | no selection |
| #7 | Method: | clinical trial |
| #8 | Author/Association: | no limited |
| #9 | Title Only: | no limited |
| #10 | Source: | no limited |
| #11 | Published Since: | no limited |
| #12 | New records added since: | no limited |
| #13 | Score of at least: | no limited |
| #14 | Return: | 50(records at a time) |
| #15 | When searching: | match all search terms (AND) |
